# LPLUNC1 Inhibits Nasopharyngeal Carcinoma Cell Growth via Down-Regulation of the MAP Kinase and Cyclin D1/E2F Pathways

**DOI:** 10.1371/journal.pone.0062869

**Published:** 2013-05-01

**Authors:** Yixin Yang, Qianjin Liao, Fang Wei, Xiaoling Li, Wenling Zhang, Songqing Fan, Lei Shi, Xiayu Li, Zhaojian Gong, Jian Ma, Ming Zhou, Juanjuan Xiang, Shuping Peng, Bo Xiang, Hao Deng, Yunbo Yang, Yong Li, Wei Xiong, Zhaoyang Zeng, Guiyuan Li

**Affiliations:** 1 Hunan Key Laboratory of Nonresolving Inflammation and Cancer, Disease Genome Research Center, The Third Xiangya Hospital, Central South University, Changsha, Hunan, China; 2 Key Laboratory of Carcinogenesis of Ministry of Health and Key Laboratory of Carcinogenesis and Cancer Invasion of Ministry of Education, Cancer Research Institute, Central South University, Changsha, Hunan, China; 3 Hunan Provincial Tumor Hospital and the Affiliated Tumor Hospital of Xiangya School of Medicine, Central South University, Changsha, Hunan, China; 4 The Second Xiangya Hospital, Central South University, Changsha, Hunan, China; 5 Department of Biochemistry and Molecular Biology, School of Medicine, University of Louisville, Louisville, Kentucky, United States of America; H. Lee Moffitt Cancer Center & Research Institute, United States of America

## Abstract

Long-palate, lung and nasal epithelium clone 1 (LPLUNC1) gene expression is relatively tissue specific. It is highly expressed in nontumor nasopharyngeal epithelial tissues, but its expression is reduced in nasopharyngeal carcinoma (NPC), indicating that LPLUNC1 may be associated with the tumorigenesis of NPC. To study the effects of LPLUNC1 on NPC tumorigenesis, a full-length LPLUNC1 expression plasmid was stably transfected into the NPC cell line, 5-8F. Our data indicated that LPLUNC1 inhibited NPC cell proliferation *in vitro* and tumor formation *in vivo*. LPLUNC1 also delayed cell cycle progression from G1 to S phase and inhibited the expression of cyclin D1, cyclin-dependent kinase 4 (CDK4) and phosphorylated Rb. To further investigate the molecular mechanisms underlying the suppressive effects of LPLUNC1 on NPC tumorigenesis, cDNA microarray was performed. These studies revealed that LPLUNC1 inhibited the expression of certain mitogen-activated protein (MAP) kinases (MAPK) kinases and cell cycle-related molecules. Western blotting confirmed that the expression of MEK1, phosphorylated ERK1/2, phosphorylated JNK1/2, c-Myc and c-Jun were inhibited by LPLUNC1. Furthermore, the transcriptional activity of AP-1 was down-regulated by LPLUNC1, suggesting that the MAPK signaling pathway is regulated by LPLUNC1. Taken together, the present study indicates that LPLUNC1 delays NPC cell growth by inhibiting the MAPK and cyclin D1/E2F pathways and suggests that LPLUNC1 may represent a promising candidate tumor suppressor gene associated with NPC.

## Introduction

Nasopharyngeal carcinoma (NPC) is an epithelial cancer with a remarkable geographic and racial distribution worldwide. Mounting evidence suggests that NPC tumorigenesis could result from environmental conditions and genetic factors [1], [2]. Tumorigenesis is a multi-step and multi-factor process that commonly involves the inactivation of several tumor suppressor genes and abnormal activation of several oncogenes. Comparative genomic hybridization and loss of heterozygosity have provided a comprehensive view of the gross genomic changes linked with NPC and revealed several new sites of genomic imbalance, indicating the possible involvement of novel oncogenes/tumor suppressor genes in the carcinogenesis of NPC [3]–[8]. However, the mechanisms of NPC tumorigenesis remain unclear.

In our previous study, we performed suppression subtractive hybridization (SSH) and cDNA microarray hybridization of NPC biopsies and nontumor nasopharyngeal epithelial tissues. We identified two PLUNC (palate lung and nasal epithelium clone protein) family members, SPLUNC1 (Short palate, lung and nasal epithelium clone 1) and LPLUNC1 (long-palate, lung and nasal epithelium clone 1) that were down-regulated in NPC, suggesting that the abnormal expression of SPLUNC1 and LPLUNC1 may be important molecular events in NPC development [9].

SPLUNC1 and LPLUNC1 are members of the PLUNC family, which is predominantly expressed in the upper airways, nose and mouth, and they are located on a single locus on chromosome 20 [10]. The PLUNC proteins are secreted and categorized into short (SPLUNC) and long (LPLUNC) forms, which contain domains that are structurally similar to one or two domains of protein/lipid binding protein (BPI/LBP), respectively [11]. SPLUNC1 was originally found to be down-regulated in NPC and exhibits host defense properties [9]. SPLUNC1 is secreted by large airway epithelial cells and has been shown to possess antimicrobial and anti-inflammatory functions [11]. LPLUNC1 belongs to the bactericidal permeability increasing BPI/LBP family, and its expression is reduced in NPC [9]. However, the function of LPLUNC1 in NPC remains unknown. In the present study, we confirmed that LPLUNC1 expression was reduced in NPC samples. Using a NPC cell line stably expressing LPLUNC1, we provided evidence that LPLUNC1 negatively regulates cell proliferation in NPC, which might be achieved by inhibition of the mitogen-activated protein (MAP) kinase (MAPK) and cyclin D1/E2F pathways.

## Materials and Methods

### Ethics Statement

Before study initiation, ethical approval was obtained from the Cancer Hospital of the Hunan province in Changsha and the Central South University Ethics Review committees/Institutional Review Boards. NPC samples, nontumor nasopharyngeal epithelial tissues and peripheral blood lymphocytes from normal volunteers were collected at the Cancer Hospital of the Hunan province. Written informed consent was obtained from all patients and volunteers.

All animal procedures were conducted in accordance with protocols approved by the Institutional Animal Care and Use Committee (IACUC) of Central South University. Animals were allowed access to standard chow diet and water ad libitum and were housed in a pathogen-free barrier facility with a 12L:12D cycle. The mice were sacrificed by CO2 asphyxiation.

### Clinical Samples and Tissue Array

NPC samples and nontumor nasopharyngeal epithelial tissues were collected at the Cancer Hospital of the Hunan province in Changsha with the approval of hospital and personal authorities. All NPCs were defined as nonkeratinizing carcinoma (WHO II). The nontumor nasopharyngeal epithelia were collected from independent patients with chronic inflammation of nasopharyngeal mucosa. Tissue array was constructed as described in our previous study [12]. Fifty-three chronic inflammations of nasopharyngeal mucosa and 192 nonkeratinizing carcinomas (NPC) were placed in one TMA block.

### RNA Isolation, Northern Blotting, and Real-time PCR Analysis

Total RNA was isolated from the cells or tissues using TRIzol reagent (Invitrogen, Breda, Netherlands). Nonisotopic Northern blotting was performed using a NorthernMax kit (Ambion, Inc. Austin, Texas, USA) according to the manufacturer's instructions. A reverse transcription reaction was performed using a Fermentas Revert Aid First Strand cDNA Synthesis kit (Fermentas, Glen Burnie, Maryland, USA) according to the manufacturer's instructions. Real-time quantitative PCR (qPCR) was performed using an IQ5 Multicolor Detection System (Bio-Rad, Berkeley, California, USA), and glyceraldehyde-3-phosphate dehydrogenase (GAPDH) was used as an internal control. The relative changes in gene expression were calculated using the 2^−ΔΔ^CT (where CT is threshold cycle) method. Three replicates were performed in parallel for each sample in each experiment, and the results are expressed as the mean of three independent experiments. The primer pairs for amplification of the LPLUNC1 and GAPDH genes are as follows: 5′- GTTGGACTCACAGGGAAAGG-3′ and 5′-GCACTCTCAGGAAGCACAGA-3′ and 5′-CAACGGATTTGGTCGTATTGG -3′ and 5′-TGACGGTGCCATGGAATTT-3′, respectively.

### Immunohistochemistry

Immunohistochemistry (IHC) was performed using the peroxidase antiperoxidase technique after a microwave antigen retrieval procedure [13], [14]. The sections were incubated with mouse anti-human LPLUNC1 (1∶200, Abcam, Cambridge, Massachusetts, USA), extracellular signal-regulated kinase (ERK), c-Jun NH2-terminal protein kinase (JNK), c-MYC, c-Jun, cyclin-dependent kinase 4 (CDK4) or p27 (Cell Signaling, Danvers, Massachusetts, USA) antibodies overnight at 4°C. A semiquantitative scoring criterion for IHC was used, in which both staining intensity and positive areas were recorded.

### Chemicals, Cell Culture, Plasmids and Stable Transfection

Lipopolysaccharides (LPS) from *Escherichia coli* 0111:B4 were purchased from Sigma (St. Louis, Missouri, USA), and mitogen-activated protein kinase kinase (MEK) inhibitor U0126 was purchased from the Cell Signaling Technology, Inc (Danvers, Massachusetts, USA). The human NPC cell line, 5-8F, was obtained from the Cancer Research Institute of Sun Yatsen University (Guangzhou, China) [15]. 5-8F cells were cultured in RPMI 1640 medium (Invitrogen, Breda, Netherlands) supplemented with 10% FCS, 100 U/ml penicillin and 100 µg/ml streptomycin. LPLUNC1 cDNA was amplified from the human cDNA library. The GFP-C2 vector (BD Clontech, Franklin Lakes, New Jersey, USA) was used to construct the LPLUNC1 expression vector, which encoded a fusion protein containing GFP and LPLUNC1. The pCMV-myc-LPLUNC1 expression plasmid was constructed using the same methods. The promoter of the cyclin D1 gene was amplified by PCR and cloned as a 1.5-kb fragment in front of the luciferase gene in the PGL3-enhancer vector. For construction of the E2F or AP-1 responsive luciferase reporters, synthetic oligonucleotides containing four tandem E2F or AP-1 binding sites as well as mutants (negative control) were ligated in front of the luciferase gene in the PGL3-enhancer vector. The sequences of the synthetic oligonucleotides are as follows: E2F wild type, ttttcGCGCttaaatta tttaagcgcGAAAacta ttttcGCGCttaaatta tttaagcgcGAAAacta; E2F mutation, ttttcatatttaaatta tttaagcgcatttacta ttttcatatttaaatta tttaagcgcatttacta; AP-1 wild type, agcTGACtaatga agcTGACtaatga agcTGACtaatga agcTGACtaatga; and Ap-1 mutation, agcgctttaatga agcgctttaatga agcgctttaatga agcgctttaatga.

Stable transfection was performed with Lipofectamine (Invitrogen, Breda, Netherlands) following the low serum protocol provided by the manufacturer. A total of 2 µg of plasmid was used in each transfection experiment. Transfected cells were cultured in complete medium for 48 h and then selected for three weeks in medium containing 800 µg/ml G418/Geneticin (Life Technologies, Grand Island, New York, USA) and routinely maintained in a medium containing 250 µg/ml G418. Expression levels of LPLUNC1 in control (vector) and LPLUNC1 transfected cells were determined using Western blot analysis with an anti-GFP antibody (Santa Cruz Biotechnology, Dallas, Texas, USA).

### MTT, Growth Curve Assay, Colony Formation Assay and BrdU Staining

For MTT assays, 1×10^4^ 5-8F cells were seeded into 96-well plates and cultured for 72 h. A total of 10 µl MTT (5 mg/ml) was added to each well, and the plates were read on a Dynatech EL309 Microelisa reader using a wavelength of 570 nm with a reference wavelength of 450 nm.

For growth curve assays, 1×10^4^ cells were seeded into 24-well plates, and the number of cells were counted with a hemocytometer every 24 h. Colony formation and soft-agar assays were performed as previously described [16]. Colonies were counted manually, imaged by microscopy and photographed after two weeks. The number of colonies per plate in the colony formation assay was calculated from the average of three independent experiments with duplicate samples in each experiment. The ability of the cells to form macroscopically visible colonies in soft agar was determined according to the standard protocol.

For BrdU staining, 2×10^5^ cells were seeded into each well of a 6-well plate containing pre-placed coverslips. A total of 8 hours later, BrdU was added to achieve a final BrdU concentration of 30 nM. Sixteen hours later, cells were fixed in methanol/acetone and processed for BrdU staining using a primary BrdU antibody (Santa Cruz Biotechnology, Dallas, Texas, USA). BrdU-positive nuclei were visualized by diaminobenzidine staining (brown), and the nuclei were highlighted with a hematoxylin counterstain (blue). A total of 500–1,000 nuclei were counted under a microscope. All of the assays were repeated three times.

### Flow Cytometry Analysis of Cell Cycle Distribution and Cyclin Expression

To assess the cell cycle distribution, cells were collected, washed with PBS and fixed in 70% (v/v) ethanol overnight. Cells were centrifuged at 1,000 g for 10 min, resuspended in 50 µg/ml propidium iodide (Sigma, St. Louis, Missouri, USA) and then immediately subjected to flow cytometry analysis on a FACStar (Becton-Dickinson, Mountain View, California, USA). Approximately 10,000 cells were examined for each sample, and the data were analyzed with CELLQuest software (BD Biosciences, Franklin Lakes, New Jersey, USA).

To analyze cyclin expression, cells were grown to subconfluence, harvested, washed extensively with PBS and then fixed and incubated with monoclonal antibodies against cyclin D1, A, E or B (Santa Cruz Biotechnology, Dallas, Texas, USA) overnight at 4°C. The cells were incubated with appropriate FITC-conjugated secondary antibody for 45 min at 37°C in the dark and analyzed for flow cytometry using a FACStar (Becton-Dickinson, Mountain View, California, USA). For each sample, data were collected from 10,000 cells. For the control, cells were incubated with secondary antibody alone to reveal background fluorescence levels.

### Tumor Formation Assay in Nude Mice

Male BALB/c nude mice at 6–8 weeks of age were used in the experiment. The mice were divided into two groups with three mice per group. To assess the effect of LPLUNC1 on tumorigenicity *in vivo*, 2×10^6^ 5-8F/vector or 5-8F/LPLUNC1 stably transfected cells were subcutaneously injected in each flank of the mice. The mice were weighed, and three dimensions of the tumors were measured every four days. The mice were sacrificed after 40 days. Tumor volume was estimated by the following equation: 4π/3*(L/2*W/2*H/2; L = length, W = width, H = height of tumor). The animal experiments were performed in accordance with the institutional guidelines.

### cDNA Microarray Analysis

Total RNA was isolated from 5-8F/vector and 5-8F/LPLUNC1-transfected cells using Trizol reagent and treated with DNase I to eliminate possible genomic DNA contamination. The whole human genome cDNA microarray provided by Biostar Gene (Biostar Gene Technology Co Ltd, Shanghai, China) was used for hybridization [12]. Three-microgram aliquots of RNA from 5-8F/vector and 5-8F/LPLUNC1-transfected cells were labeled with Cy5-dCTP and Cy3-dCTP, respectively. The hybridization, washing, and scanning were performed as previously described [17]. A ratio of down−/up-regulation ≥2 was considered to be significant.

### Transient Transfection and Luciferase Analysis

For luciferase analysis, 5×10^4^ cells were plated into each well of a 12-well plate and incubated for 12 h prior to transfection. Transfections were performed with Lipofectamine according to the manufacturer’s instructions (Invitrogen, Breda, Netherlands). For each transfection, 2 µg reporter plasmid and 4 µg pCMV-myc-LPLUNC1 plasmid or empty vector were cotransfected into 5-8F cells with 0.5 µg β-galactosidase expression vector as an internal control (Promega, Madison, Wisconsin, USA). All experiments were performed three times. Cell lysates were collected 48 h post-transfection and assayed for luciferase activity using an enhanced luciferase assay kit (Promega, Madison, Wisconsin, USA). The results were normalized to β-galactosidase activity.

### Western Blotting and Kinase Assay

Cells from 100-mm culture dishes were lysed with 200 µl 1× SDS and sonicated for 15 sec. Lysates (30 µl) were resolved on 10% SDS-PAGE gradient gels. The membrane was incubated with a GFP mouse monoclonal antibody, MEK1 (Santa Cruz Biotechnology, Dallas, Texas, USA), ERK, phosphorylated ERK, JNK, phosphorylated JNK, c-Jun, phosphorylated Rb, cyclin D1, or CDK4 antibodies (Cell Signaling, Danvers, Massachusetts, USA) overnight at 4°C. Immunobands were detected using Supersignal West Extended Duration Substrate (Pierce, Rockford, Illinois, USA). An antibody against α-tubulin (Santa Cruz Biotechnology, Dallas, Texas, USA) served as an endogenous control.

The kinase assay for ERK1/2 was performed with a p44/42 MAP kinase assay kit (Cell Signaling, Danvers, Massachusetts, USA) according to the manufacturer’s protocol. Briefly, sepharose bead conjugated phospho-p44/42 MAPK monoclonal antibody was used for selective immunoprecipitation (IP) of active phospho-p44/42 MAP kinase from cell extracts. Next, IP pellets were incubated in kinase buffer containing ATP and recombinant Elk-1 (as MAP kinase substrate). Finally, MAP kinase activity was measured by detecting MAP kinase-induced phosphorylation of Elk-1 using phosphor-ELK-1 antibody and Western blotting.

### Statistical Analysis

Correlations between different clinical status and LPLUNC1 positive expression were analyzed using a χ^2^ test. All the data, except for the tissue array analysis, were analyzed by Student’s t-test using the SPSS program. P-values less than 0.05 were considered statistically significant.

## Results

### Expression of LPLUNC1 in NPC Biopsies

**Figure 1:**
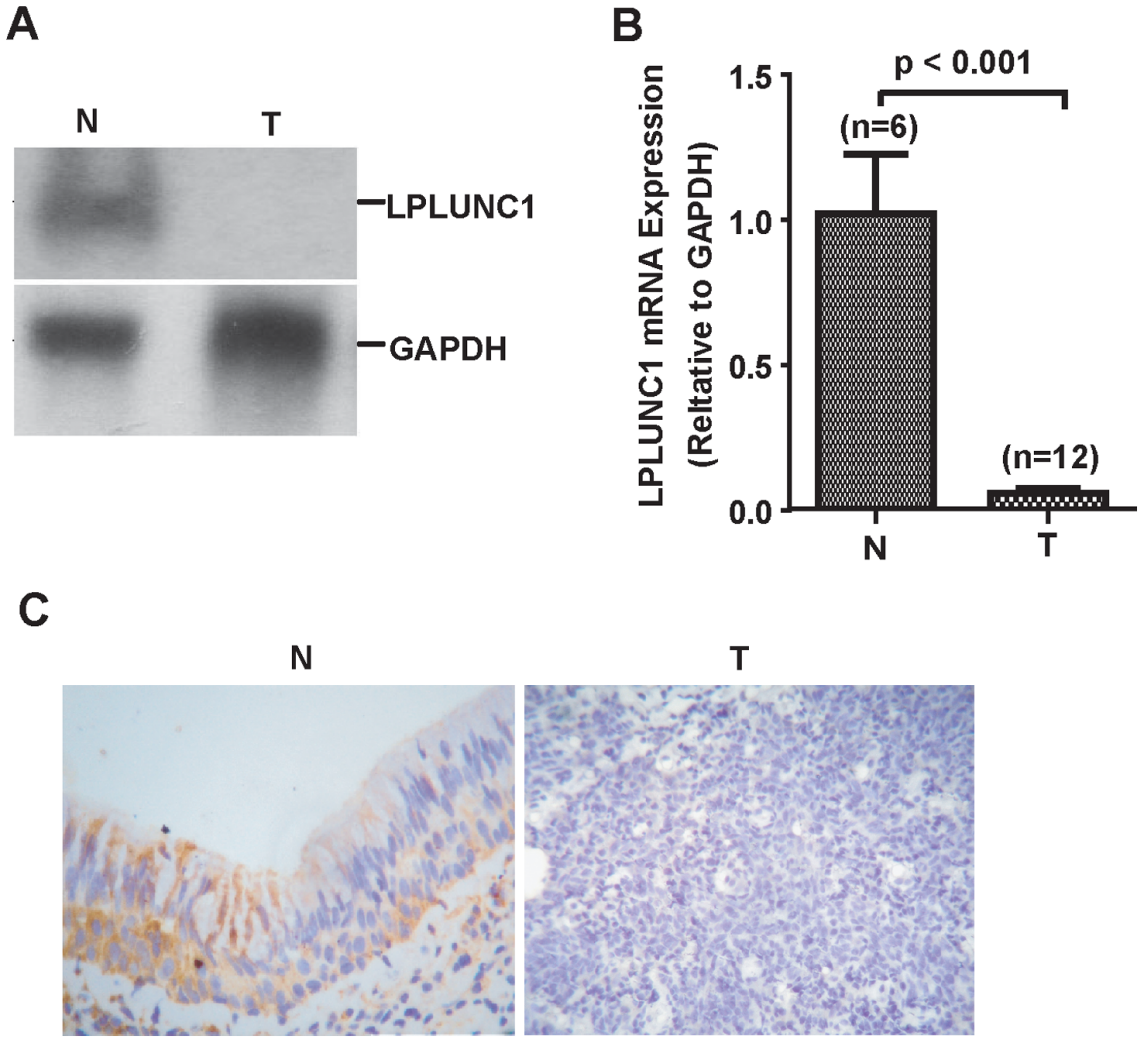
The expression of LPLUNC mRNA in nontumor nasopharyngeal epithelial tissues and NPC biopsies. LPLUNC1 was highly expressed in nontumor nasopharyngeal epithelial tissues but was absent or expressed at low levels in NPC biopsies. N, nontumor nasopharyngeal epithelial tissues; T, NPC biopsies. The housekeeping gene, GAPDH, was used as an internal control. A. Expression of LPLUNC mRNA in nasopharyngeal epithelial and NPC samples was detected by Northern blotting. B. Expression of LPLUNC mRNA in nasopharyngeal epithelial and NPC samples was measured by RT-PCR; p<0.001. C. Expression of LPLUNC1 protein in nasopharyngeal epithelial and NPC samples was measured by immunohistochemistry; p<0.001.

Our previous data showed that LPLUNC1 was specifically expressed in nasopharyngeal epithelial tissue and the trachea, whereas its expression was reduced in NPC biopsies [9]. To explore whether LPLUNC1 is involved in the function of NPC, we confirmed its expression in one nontumor nasopharyngeal epithelial tissue and one nonkeratinizing NPC by Northern blotting. As shown in Figure 1A, LPLUNC1 mRNA was not detectable in the NPC sample, whereas the nontumor nasopharyngeal epithelial tissue displayed a high level of LPLUNC1 mRNA, indicating that LPLUNC1 expression was reduced in NPC. Real-time PCR analysis was used to confirm the level of LPLUNC1 expression in 12 nonkeratinizing nasopharyngeal carcinomas compared with 6 nontumor nasopharyngeal epithelial tissues. The data showed that LPLUNC1 mRNA was not detectable or expressed at low levels in NPC samples but was highly expressed in nontumor nasopharyngeal epithelial tissues (p<0.001, Figure 1B).

Meanwhile, a tissue array of NPC was used to examine the expression of LPLUNC1 at the protein level by immunohistochemistry. LPLUNC1 protein was expressed in 27.3% (36/192) of the NPC patients, whereas 75.4% (40/53) of the non-NPC patients were found to express LPLUNC1 (Figure 1C). Statistical analysis using a χ^2^ test revealed that the non-NPC group had a higher frequency of LPLUNC1 expression than the NPC groups.

### Over-expression of LPLUNC1 Inhibits NPC Cell Growth and Proliferation *in vitro*

**Figure 2:**
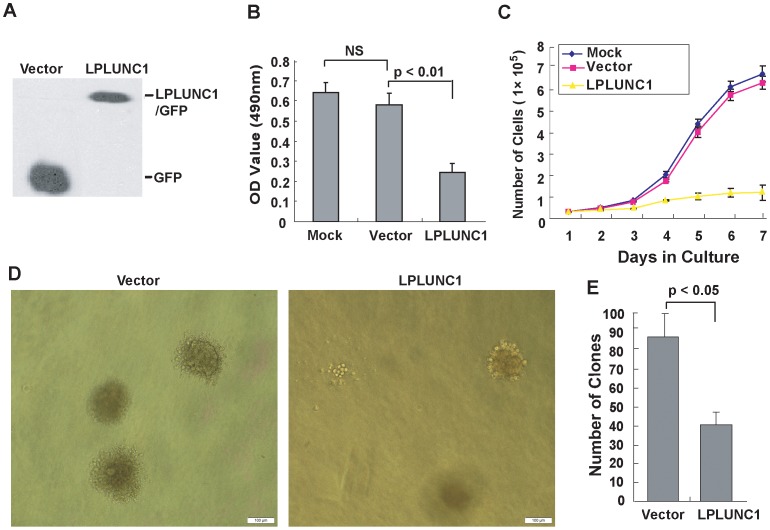
**LPLUNC1 inhibits 5-8F NPC cell growth and proliferation.** A. Western blot analysis of LPLUNC1 expression in 5-8F/vector and 5-8F/LPLUNC1 cells using the anti-GFP primary antibody revealed that 5-8F/LPLUNC1 cells expressed the GFP tagged LPLUNC1 protein, whereas 5-8F/vector cells expressed GFP protein alone. B. MTT assay of 5-8F/LPLUNC1 cells. A total of 1×10^4^ cells were seeded into 96-well, flat-bottom plates and cultured for 72 h, and 10 µl of MTT (5 mg/ml) was added. LPLUNC1 expression significantly inhibited cell proliferation; p<0.01. C. Growth curve of the LPLUNC1 stably transfected 5-8F cells. A total of 1×10^4^ cells were seeded into 24-well plates, and the number of cells were counted with a hemocytometer every 24 h. The expression of LPLUNC1 markedly inhibited cell growth; p<0.01. D. Soft agar colony formation assay of 5-8F/LPLUNC1 cells. Colonies were manually counted and imaged using microscopy and photographed two weeks later (magnification = 100×, Bar = 100 µm). E. The number of colonies per plate in the colony formation assay was derived from three independent experiments, with duplicates in each experiment. The data are presented as the mean ± SD; p<0.05. Both the size and number of the colonies formed in soft agar were decreased in 5-8F/LPLUNC1 cells compared with 5-8F/vector cells.

We reasoned that if loss of LPLUNC is an important event for cancer progression in NPC, then re-expression of LPLUNC1 would inhibit cancer cell growth and slow tumor development. We selected a poorly differentiated nasopharyngeal squamous carcinoma cell line (5-8F) to stably transfect with pEGFP-C2 (empty vector) and pEGFP-C2-LPLUNC1 (LPLUNC1) plasmids. The expression level of LPLUNC1 in 5-8F/empty vector- and 5-8F/LPLUNC1-transfected cells was determined by Western blot analysis using an anti-GFP antibody. Our data showed that the expression of LPLUNC1 in 5-8F/LPLUNC1-transfected cells was significantly enhanced (Figure 2A).

Next, we performed MTT assays to investigate the role of LPLUNC1 on 5-8F cell proliferation. In total, 1×10^4^ 5-8F/mock-, 5-8F/vector- and 5-8F/LPLUNC1-transfected cells were seeded into 96-well plates and grown for 4 days. Cell proliferation was markedly attenuated in the cell line stably expressing LPLUNC1 compared with the vector control (p<0.01, Figure 2B).

Next, we checked whether up-regulation of LPLUNC1 could inhibit cancer cell growth and proliferation in 5-8F cells. As shown in Figure 2C, cell growth was markedly decreased in 5-8F/LPLUNC1-transfected cells compared with 5-8F/vector-transfected cells. After 7 days, the number of 5-8F/LPLUNC1 cells was 5-fold lower than that of the 5-8F/vector cells (p<0.01).

Soft agar assays were performed to assess the colony formation ability of 5-8F/LPLUNC1- and 5-8F/vector-transfected cells. Twenty thousand cells were seeded into a 60 mm dish, and 14 days later, the number of colonies was counted. In 5-8F/vector cells, the average number of colonies was 80±5/plate, whereas the number of colonies was only 37±6 (p<0.05) in the LPLUNC1-transfected cells. The expression of LPLUNC1 led to a significant reduction in both the rate of colony formation and the size of the colonies. Cells expressing LPLUNC1 grew slowly, whereas the matched control grew into visible cell aggregates (Figures 2D and 2E).

### Over-expression of LPLUNC1 Inhibits NPC Cell Tumorigenicity *in vivo*

**Figure 3:**
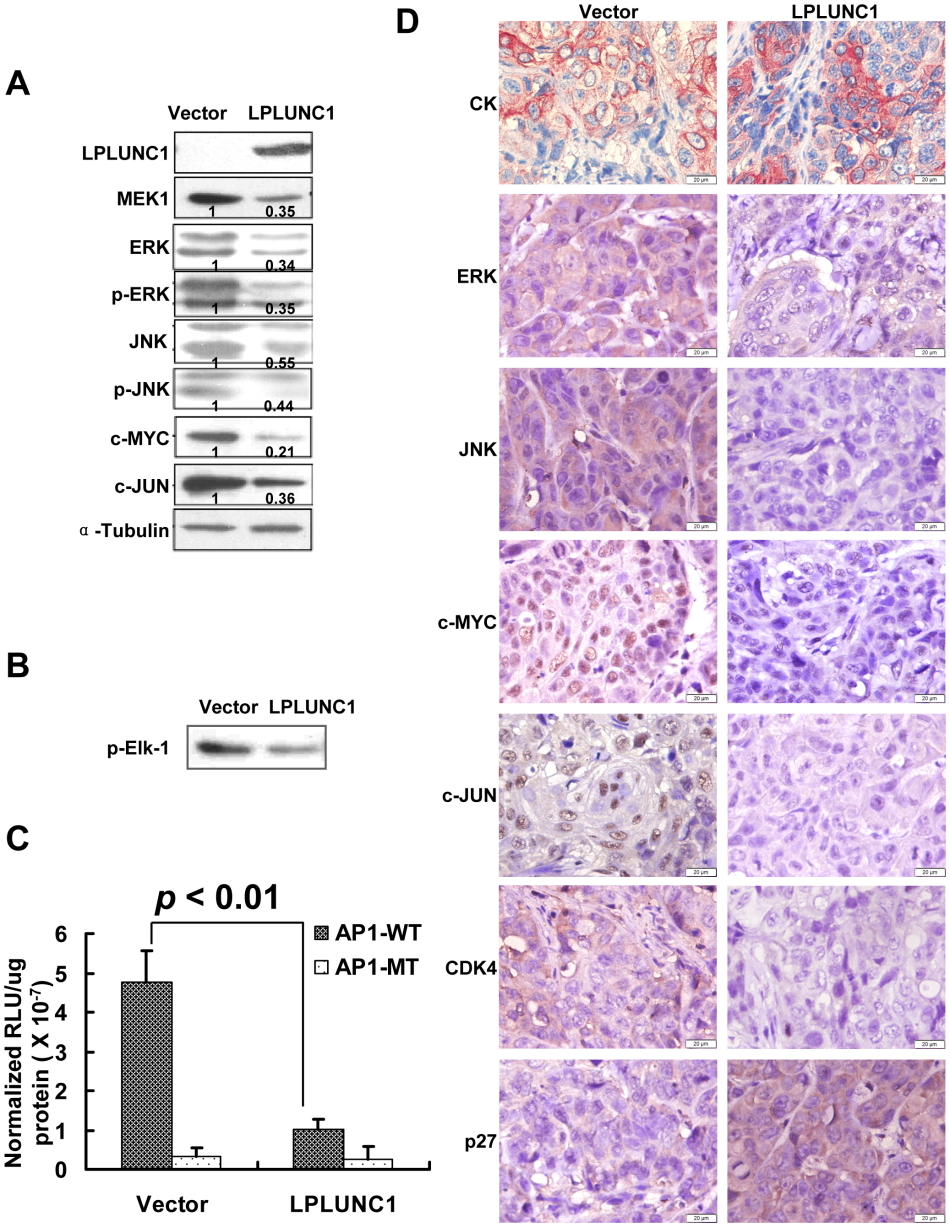
Over-expression of LPLUNC1 inhibited NPC tumor formation *in vivo*. A. Tumor formation in nude mice. The tumors that formed following injection of 2×10^6^ cells were much smaller in the 5-8F/LPLUNC1 group than tumors from the 5-8F/vector group after 40 days. B. Average tumor volume. The average volume of tumors in the 5-8F/LPLUNC1 group was significantly decreased compared with the 5-8F/vector group. The data are presented as the mean ± SD; *p<0.01.

To directly evaluate the role of LPLUNC1 in tumor formation *in vivo*, 2×10^6^ 5-8F/vector and 5-8F/LPLUNC1 cells were injected into nude mice, with each group consisting of three mice. A tumor was first detected at day 10 post-injection. The tumor volume was measured every four days, and forty days later, the mice were sacrificed. A significant reduction in tumor size was observed in the group of mice injected with LPLUNC1-overexpressing cells (Figure 3A). Tumors derived from 5-8F/vector cells were five times larger than those from LPLUNC1-expressing 5-8F cells (p<0.01, Figure 3B). Thus, our data demonstrate that LPLUNC1 suppressed cell proliferation and tumor formation of the 5-8F NPC cell line *in vivo*.

### LPLUNC1 Delays Cell Cycle Progression

**Figure 4:**
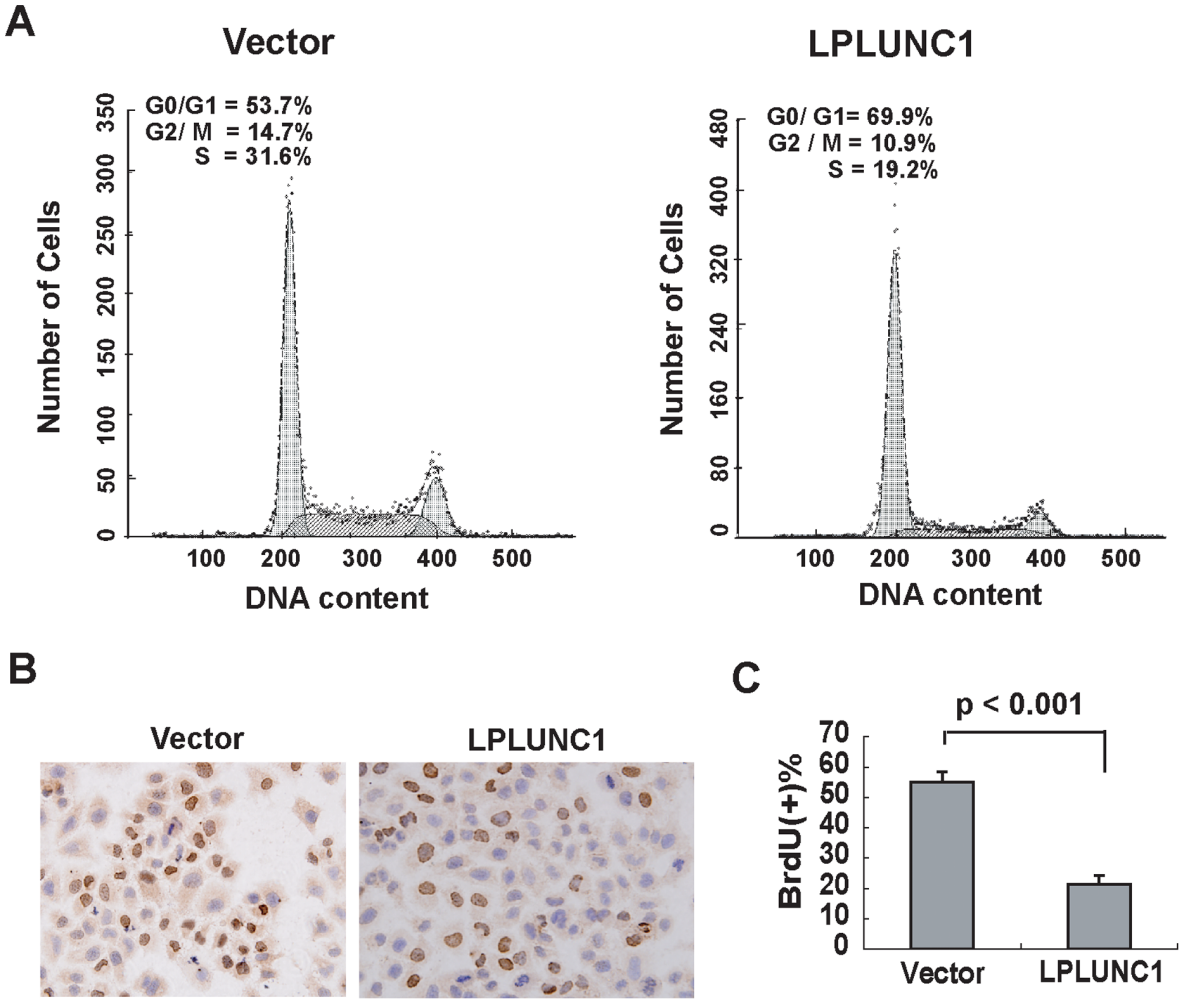
Over-expression of LPLUNC1 decreased 5-8F cell proliferation. A. Flow cytometry analysis showed that LPLUNC1 transfection increased the proportion of cells in G1 phase from 53.7% to 69.9% and decreased the proportion of cells in S phase from 31.6% to 19.2%; p<0.01. B and C. LPLUNC1 transfection decreased the number of cells positive for BrdU staining; p<0.01. Cells were treated with 30 nM BrdU for 16 h. Fixation and immunocytochemistry were conducted to assess BrdU incorporation.

Because the over-expression of LPLUNC1 inhibited cell growth and proliferation of 5-8F cells *in vitro* and *in vivo*, we investigated whether cell cycle regulation was one of the mechanisms by which LPLUNC1 exerted its growth-suppressive effect. The total cellular DNA content and the proportion of vector-transfected and LPLUNC1 stably transfected cells in various phases of the cell cycle were quantified by flow cytometry analysis. Scatter profiles and the percentage of cells in the various phases of the cell cycle showed that LPLUNC1 led to a substantial increase in the number of cells in G0-G1 phase (53.67±3.06% for 5-8F/vector and 69.93±3.48% for 5-8F/LPLUNC1 cells) and a reduction in the number of cells in S phase (31.57±1.96% for 5-8F/vector and 19.17±1.65 for 5-8F/LPLUNC1 cells). LPLUNC1 expression had little effect on the number of cells in G2-M phase (Figure 4A). Furthermore, the number of BrdU-positive cells was decreased in 5-8F/LPLUNC1 cells (21.5±2.89) compared with 5-8F/vector cells (55.00±3.56); this finding was consistent with the results of our flow cytometry analysis (Figures 4B and 4C).

### LPLUNC1 Regulates the Cell Cycle of 5-8F Cells via the MAP Kinase Pathway

To identify the mechanism by which LPLUNC1 regulates cell growth, cDNA microarrays were employed to screen for genes regulated by LPLUNC1. A total of 369 genes were found to be differentially regulated by the expression of LPLUNC1 (ratio of expression ≥2).

To identify signaling pathways that are associated with LPLUNC1, we analyzed the 369 differentially expressed genes using the DAVID software. The results showed that multiple upstream activators of the “classical” mitogen-activated protein kinase (MAPK) and MAPK kinases, such as N-RAS, RASSF4, MAP4K5, MAP3K1, MAP4K4, MAP3K8, MAP3K7 and MAPK9, and cell cycle molecules, including CCNC, CDH2, CDK7, CDK8 and CDKL2, were inhibited by LPLUNC1 (Table 1). Thus, the cell cycle and MAPK pathways were altered by LPLUNC1 expression in 5-8F cells.

**Table 1:** MAPK Kinase and Cell Cycle Molecules Regulated by LPLUNC1.

MAP kinases regulated by LPLUNC1			
Gene Symbol	Protein/gene definition	Genebank	Fold Change (LPLUNC/Vector)
MAP4K5	Homo sapiens mitogen-activated protein kinase kinase kinase kinase 5 (MAP4K5),transcript variant 2, mRNA	NM_198794	0.41
RASSF4	Homo sapiens Ras association (RalGDS/AF-6) domain family 4 (RASSF4), transcript variant 1, mRNA	NM_032023	0.43
NRAS	Human N-ras mRNA and flanking regions	NM_002524	0.43
MAP3K1	Homo sapiens mitogen-activated protein kinase kinase kinase 1 (MAP3K1), mRNA	NM_005921	0.43
MAP4K4	Homo sapiens mitogen-activated protein kinase kinase kinase kinase 4 (MAP4K4),transcript variant 2, mRNA	NM_145686	0.47
MAP3K8	Homo sapiens mitogen-activated protein kinase kinase kinase 8 (MAP3K8), mRNA	NM_005204	0.47
MAP3K7	Homo sapiens mitogen-activated protein kinase kinase kinase 7 (MAP3K7), transcriptvariant A, mRNA	NM_003188	0.47
MAPK9	Homo sapiens mitogen-activated protein kinase 9, transcript variant 1, mRNA (cDNAclone MGC:45322 IMAGE:5528624), complete cds	NM_002752	0.48
**Cell cycle regulated by LPLUNC1**			
E2F5	Homo sapiens E2F transcription factor 5, p130-binding (E2F5), mRNA	NM_001951	0.40
RBAK	Homo sapiens RB-associated KRAB repressor (RBAK), mRNA	NM_021163	0.42
CCNC	Homo sapiens, Similar to cyclin C, clone IMAGE:5759974, mRNA	NM_005190	0.44
CDH2	N-cadherin, human, umbilical vein endothelial cells, mRNA	NM_001792	0.44
GRB2	Homo sapiens growth factor receptor-bound protein 2 (GRB2), transcript variant 1	NM_002086	0.45
CDK7	Homo sapiens cyclin-dependent kinase 7 (MO15 homolog, Xenopus laevis, cdk-activating kinase) (CDK7), mRNA	NM_001799	0.46
CDKL2	Homo sapiens cyclin-dependent kinase-like 2 (CDC2-related kinase) (CDKL2), mRNA	NM_003948	0.46
CDK8	Homo sapiens cyclin-dependent kinase 8 (CDK8), mRNA	NM_001260	0.50
CDC42SE1	Homo sapiens CDC42 small effector 1 (CDC42SE1), mRNA	NM_020239	2.22
CDKN1B	Homo sapiens cyclin-dependent kinase inhibitor 1B (p27, Kip1) (CDKN1B), mRNA	NM_004064	2.03

**Figure 5:**
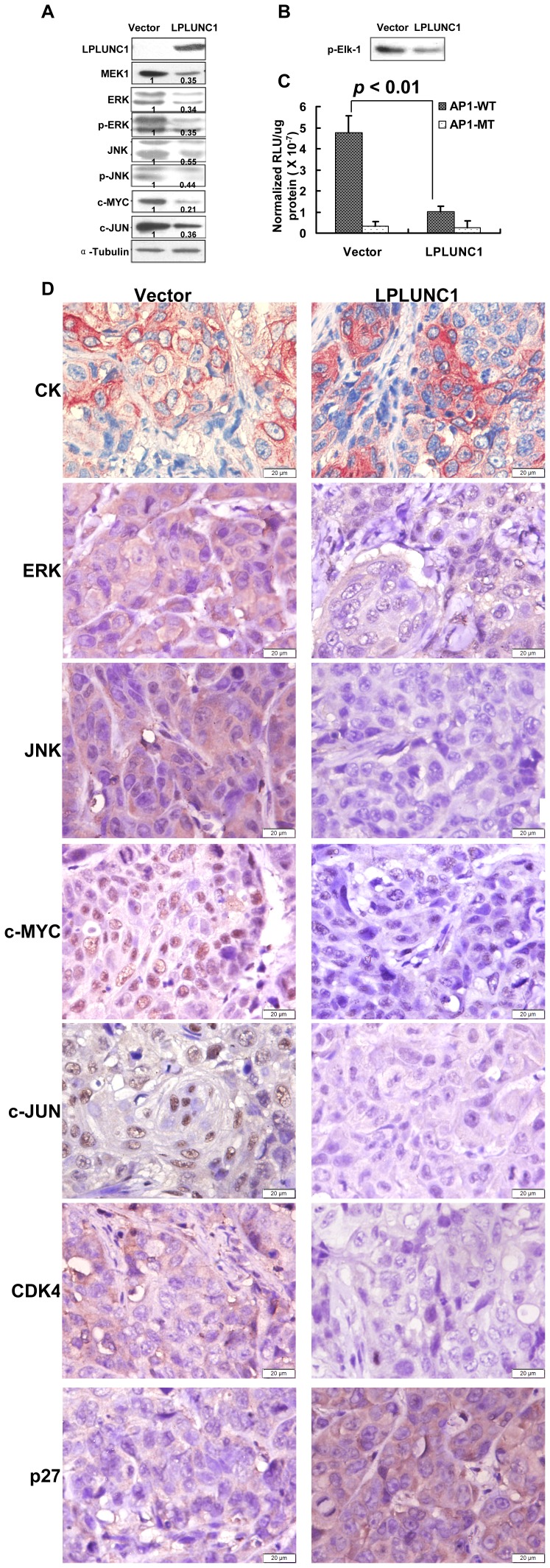
Over-expression of LPLUNC1 inhibited the expression of MAPKs and their downstream molecules in 5-8F cells. A. Western blotting analysis of MAPKs. Levels of MEK1, ERK, JNK, phosphorylated ERK, phosphorylated JNK, c-MYC and c-JUN were inhibited by LPLUNC1. α-Tubulin served as a loading control. The data shown are representative of at least three separate experiments. Quantification of protein levels by densitometric analyses of band intensities are marked under each band. B. ERK1/2 kinase activity was measured by p44/42 MAP kinase assay kit. Elf-1 phosphorylation was detected using phosphor-Elf-1 antibody by western blotting. The right lane in LPLUNC1-transfected cells resulted in lower ERK1/2 MAP kinase-induced phosphorylation of Elk-1 compared with the left lane in control vector transfected cells reflecting that LPLUNC1 inhibited ERK kinase activity. C. Luciferase assay measuring AP-1 transcriptional activity. The luciferase reporter plasmid containing wild type AP-1 (AP1-WT) responsive sites was co-transfected with pCMV-myc-LPLUNC1 or empty vector plasmid into 5-8F cells. The luciferase reporter plasmid containing a mutant sequence (AP1-MT) was used as a control. LPLUNC1 over-expression significantly inhibited the promoter activity of AP-1. The data are presented as the mean ± SD; p<0.01. D. Immunohistochemical staining of components of the MAPK pathway in xenograft tumor tissues showed that LPLUNC1 decreased ERK, JNK, CDK4, c-MYC and c-JUN protein expression and increased p27 expression. The epithelia cell marker cytokeratin (CK) was used to validate that xenograft tumors were poorly differentiated NPC (magnification = 400×, Bar = 20 µm).

The levels of some MAPK kinases, such as MEK1, ERK and JNK as well as phosphorylated ERK and JNK, and their downstream effector molecules, including c-MYC and c-JUN, were confirmed by Western blotting. As illustrated in Figure 5A, the levels of MEK1, phosphorylated ERK and JNK, c-MYC and c-JUN decreased in LPLUNC1-transfected cells compared with vector-transfected cells. To further validate that the MAPK pathway was regulated by LPLUNC1, we measured p44/42 MAP (ERK1/2) kinase activity in NPC cells, using Elk-1 protein as an ERK kinase substrate. The kinase assay showed that LPLUNC1 repressed ERK activity (Figure 5B).

Activator protein 1 (AP-1) is a transcription factor that is a heterodimeric protein composed of proteins belonging to the c-Jun, c-Fos, ATF and JDP families. To investigate the effect of LPLUNC1 on AP-1 transcriptional activity, the luciferase reporter plasmid containing AP-1 responsive sites was transiently co-transfected with pCMV-myc-LPLUNC1 or empty vector plasmid into 5-8F cells to measure the AP-1 transcriptional activity. As shown in Figure 5C, the AP-1 transcriptional activity was dramatically decreased in pCMV-myc-LPLUNC1 transfected-cells compared with the control.

The status of ERK, JNK, c-MYC and c-JUN were also compared in xenograft tumor tissues by immunohistochemical staining. As shown in Figure 5D, the epithelia marker cytokeratin (CK) protein was positive in xenograft tumors, and less intense of ERK, JNK, c-MYC, and c-JUN staining levels were detected in 5-8F/LPLUNC1 tumors. Together, our data clearly indicate that induction of LPLUNC1 over-expression inhibits the growth of 5-8F tumors in mice, which is associated with modulating the MAPK activation and the cell cycling event in 5-8F tumors.

### LPLUNC1 Attenuates LPS Activated MAP Kinase Pathway

**Figure 6:**
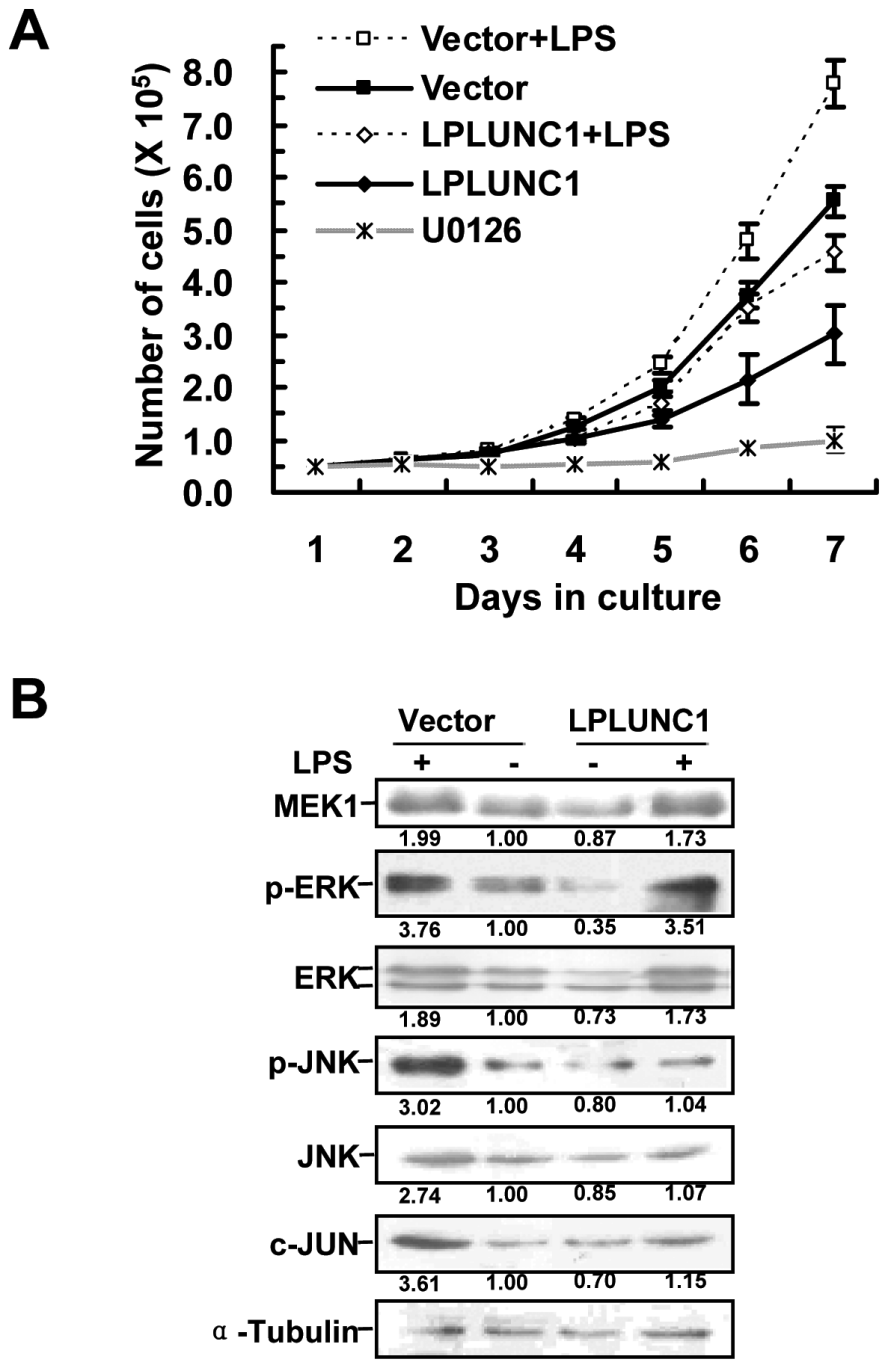
LPLUNC1 attenuates LPS activated MAP kinase pathway. A. Growth curve of 5-8F cells treated by LPS and LPLUNC1 transfection. LPS promoted NPC cell proliferation, whereas LPLUNC1 hindered LPS stimulated NPC cell proliferation. MEK1/2 inhibitor U0126 was also used to treat 5-8F cells, and the inhibition of the MAP kinase pathway by U0126 strongly decreased NPC cell growth. B. Western blotting analysis of MAPKs in 5-8F cells treated by LPS and LPLUNC1 transfection. The levels of MEK1, ERK, JNK, phosphorylated ERK and JNK, and c-JUN were increased after LPS stimulation. However LPLUNC1 inhibited the MAPK activation stimulated by LPS. Quantification of protein levels by densitometric analyses of band intensities are marked under each band.

To further validate that the MAPK pathway was being regulated by LPLUNC1, we stimulated 5-8F cells by LPS, a MAPK pathway activator [18], [19], to see whether activated MAPK pathway overrides the inhibition of proliferation engendered by LPLUNC1 or whether over-expression of LPLUNC1 attenuates the promotion of proliferation and the activation of the MAP kinase pathway by LPS. After treatment with LPS, proliferation of either LPLUNC1 or empty vector transfected 5-8F cells were accelerated (Figure 6A), and the levels of MEK1, ERK, JNK, c-JUN, and phosphorylated ERK and JNK were also increased in LPS treated 5-8F cells (Figure 6B). LPLUNC1 over-expression significantly hindered LPS stimulated NPC cell proliferation (Figure 6A) and inhibited the activation of the MAP kinase pathway components, such as JNK and c-JUN (Figure 6B). We also treated NPC cells with U0126, which is a highly selective inhibitor of MEK1/2, to confirm the function of the MAP kinase pathway in NPC cell proliferation. Inhibition of the MAP kinase pathway by U0126 strongly decreased NPC cell growth, producing the same effect as LPLUNC1-transfection (Figure 6A).

### Over-expression of LPLUNC1 Influences Cyclin Expression and Inhibits the Cyclin D1/E2F Pathway

**Figure 7:**
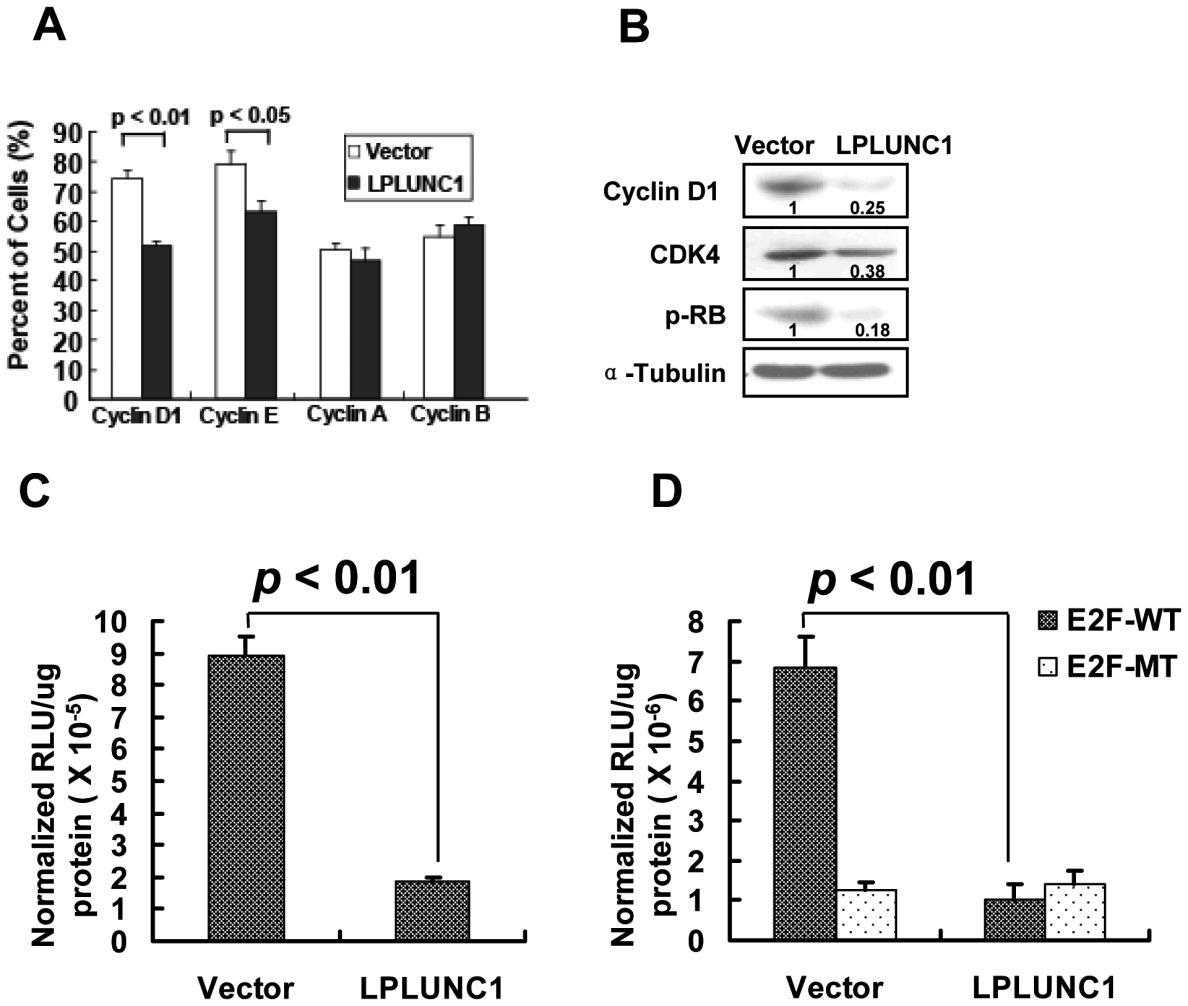
LPLUNC1 inhibited the expression of important molecules in the cell cycle pathway. A. FACS analysis of the relative expression of the cyclin family. The relative expression levels of cyclin D1 and cyclin E1 were decreased in 5-8F/LPLUNC1 cells compared with the control; p<0.01 and p<0.05, respectively. There was no difference in the expression levels of cyclin A and B between 5-8F/LPLUNC1 and 5-8F/vector cells. B. Western blotting analysis of cell cycle molecules showed that the expression levels of CDK4, cyclin D1 and phosphorylated Rb were decreased in 5-8F/LPLUNC1 compared with 5-8F/vector cells. C. and D. Luciferase assay to assess cyclin D1 promoter (C) and E2F transcriptional activity (D). The luciferase reporter plasmids containing cyclin D1 promoter (C) or E2F responsive sites (D, E2F-WT: wild type, E2F-MT: mutated) were co-transfected with pCMV-myc-LPLUNC1 or empty vector plasmid into 5-8F cells. LPLUNC1 overexpression significantly inhibited the promoter activity of cyclin D1 (C) and E2F transcriptional activity (D). The results are representative of 3 independent experiments. The data are presented as the mean ± SD; p<0.01.

To further investigate the mechanism by which LPLUNC1 delays the G0–G1 phase of 5-8F cells, we performed flow cytometry analysis for cyclin (cyclin D1, A, B and E) protein expression. The cells were subjected to indirect immunofluorescence staining using antibodies against cyclin D1, A, B and E. LPLUNC1 transfection reduced cyclin D1 and E expression compared with 5-8F/vector-transfected cells, and the expression levels of cyclin A and B were unchanged (Figure 7A).

Cyclin D1 and Rb/E2F play key roles in cell cycle progression. CDK4 is the kinase partner of cyclin D1. CDK4 and cyclin D1 can form the activated CDK4-cyclin D1 complex that promotes E2F-mediated transcription. Flow cytometry analysis indicated that the expression of cyclin D1 was attenuated in LPLUNC1-transfected cells. Moreover, Western blotting showed that the expression of CDK4, cyclin D1 and phosphorylated Rb was decreased in 5-8F/LPLUNC1 cells compared with 5-8F/vector cells (Figure 7B).

Next, transient transfection and luciferase analysis were used to measure the luciferase reporter activity under the cyclin D1 promoter or E2F responsive sites. The cyclin D1 promoter or E2F responsive sites reporter plasmids were transiently co-transfected with pCMV-myc-LPLUNC1 or an empty vector plasmid into 5-8F cells. As shown in Figures 7C and 7D, the activity of the cyclin D1 promoter and E2F transcriptional activity were significantly decreased in 5-8F cells co-transfected with pCMV-myc-LPLUNC1 compared with the control.

## Discussion

LPLUNC1 is located on chromosome 20 and belongs to the PLUNC family [10]. The PLUNC proteins are secreted and categorized into short (SPLUNC) and long (LPLUNC) forms, which contain domains that are structurally similar to one or two domains of protein/lipid binding protein (BPI/LBP), respectively [11]. It was reported that single nucleotide polymorphisms (SNPs) [20], EBV, and nanobacteria were associated with the SPLUNC1 gene in NPC [21], [22], but there are few reports about the LPLUNC1 gene in NPC. In the present study, we confirmed that LPLUNC1 was down-regulated in NPC and found that LPLUNC1 inhibited NPC cell growth *in vitro* and *in vivo*, through cell cycle arrest at G0-G1 phase; therefore LPLUNC1 may play an important role in the carcinogenesis of NPC.

Eukaryotic cell proliferation is a highly regulated system that is controlled by CDK-cyclin complexes. The cell cycle transition from the G1 to the S phase is the major regulatory checkpoint in this process. This transition is characterized by the phosphorylation of Rb, and the CDK-cyclin complex catalyzes the reaction. In this study, we found that LPLUNC1 inhibited CDK4-cyclin D1 and p-Rb, which resulted in cell cycle arrest and the exertion of its anti-proliferative effect. Cell cycle inhibitor p27 also plays an important role in the G1/S progression process. As a member of cyclin-dependent kinase inhibitors, the ability of p27 to enforce the G1 restriction point is derived from its inhibitory binding to CDK2/cylin E and other CDK/cyclin complexes. Our gene expression profiling results showed that p27 was down-regulated by LPLUNC1 at the mRNA level. Western blotting results also validated that p27 was down-regulated by LPLUNC1 at the protein level. In addition, LPLUNC1 also down-regulated cyclin E. Thus both cyclin D1 and cyclin E may perform their function in LPLUNC1 induced cell cycle arrest.

To identify the mechanism by which LPLUNC1 inhibits cell growth, we utilized cDNA microarrays to screen for potential down-stream genes as well as pathways of LPLUNC1 in NPC cells. The gene expression profiling data indicate that LPLUNC1 expression down-regulated several components of the MAP kinase pathway in NPC cells. We then focused on the repression of the MAPK pathway by LPLUNC1. MAPKs are essential components of the intracellular signal transduction pathways that regulate cell proliferation [23]. In the present study, we investigated the MAPK signaling pathway regulation by LPLUNC1. Western blotting confirmed that LPLUNC1 decreased the levels of MEK1, phosphorylated ERK and JNK, and inhibited the activation of down-stream transcriptional factors such as AP-1 and E2F. AP-1 proteins, mostly those that belong to the JUN group, control cell survival and death through their ability to regulate the expression and function of cell cycle regulators, such as cyclin D1 [24]. The E2F family of transcription factors also plays important roles in regulating the expression of cell cycle-dependent genes that are essential for G1/S transit and cellular proliferation. The MAPK pathway is downstream of Ras, but how LPLUNC1, a secreted protein, interacts with the Ras pathway and represses MAPK signaling is still unknown. Ras can also signal via PI3K and AKT to trigger cyclin D1 expression. Whether LPLUNC1 also represses cyclin D1 expression by repressing Ras signaling through PI3K/AKT should be elucidated in the future.

Human nasopharyngeal epithelium is directly exposed to the toxic dusts and pathogens of the environment. Chronic inflammation of the nasopharynx caused by these pathogens is one important cause of NPC. Bacterial LPS stimulates nasopharyngeal epithelium expressing and releasing cytokines, such as TNF-alpha, and activating MAPK signaling pathway [25]. The over-activation of the MAPK pathway leads to cell proliferation and, finally, to tumorigenesis. Bioinformatics analysis revealed that the LPLUNC1 protein has two permeability-increasing protein/lipid binding protein (BPI/LBP) domains that can bind to LPS [26]. In the present study, LPS increased the levels of components of the MAPK pathway, such as MEK1, ERK and JNK, and stimulated NPC cell proliferation, whereas LPLUNC1 superseded the promotion of proliferation by LPS. This result implied that LPLUNC1 may play an important role in anti-bacteria and prevention of NPC initiation.

In conclusion, our observations indicated that LPLUNC1, a secretive protein expressed in nasopharyngeal epithelium, may bind to bacterial LPS, inhibit LPS stimulated nasopharyngeal epithelium cell proliferation, and prevent carcinogenesis of NPC. LPLUNC1 also inhibits NPC cell growth and cell cycle progression from G1 to S phase *in vitro*, and suppresses NPC cell tumor formation *in vivo*. The most likely mechanism underlying the LPLUNC1-induced growth arrest involves down-regulation of MAP kinases, which leads to reduction in G1-related CDKs, such as CDK4 and p-RB, and a decrease in cyclin D1 and CDK4 expression, resulting in further inhibition of E2F-mediated gene transcription. However, the function of LPLUNC1 remains to be fully elucidated in NPC. A more detailed understanding of the role of LPLUNC1 in NPC carcinogenesis and progression will likely afford new opportunities for the diagnosis and treatment of this malignancy.
