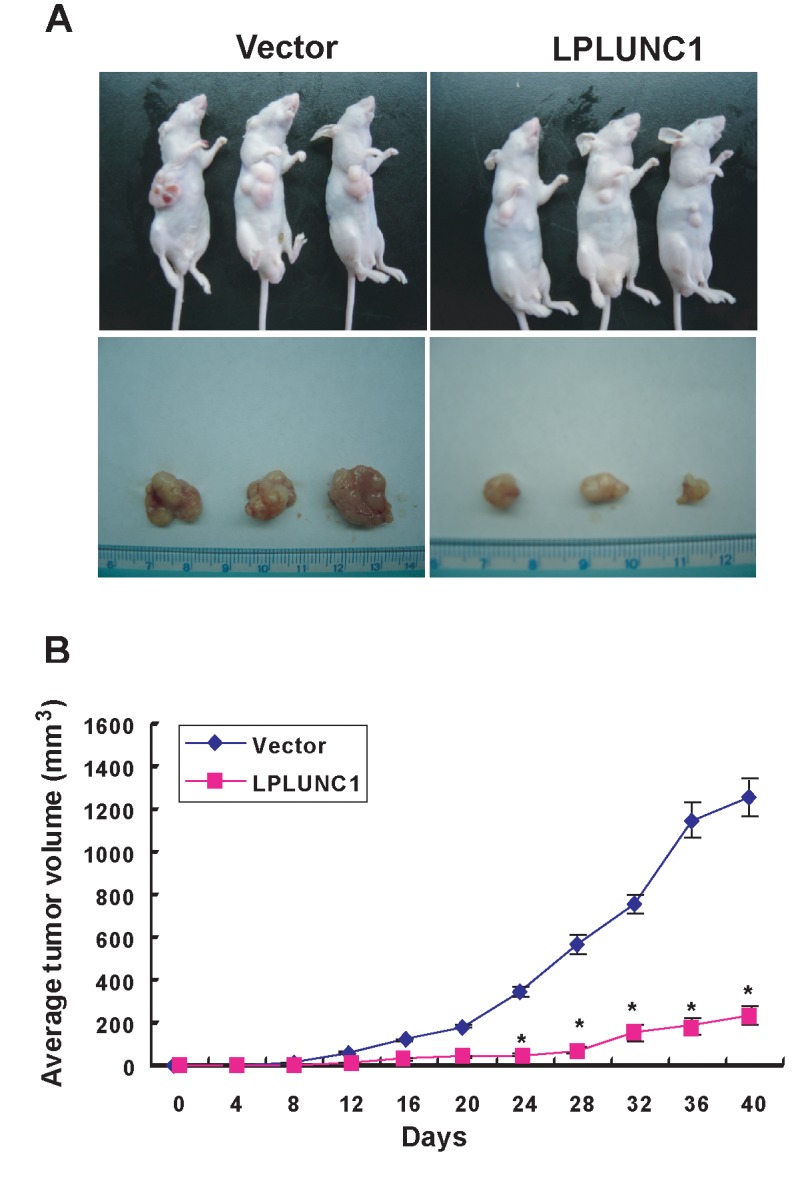# Correction: LPLUNC1 Inhibits Nasopharyngeal Carcinoma Cell Growth via Down-Regulation of the MAP Kinase and Cyclin D1/E2F Pathways

**DOI:** 10.1371/annotation/e120c2f3-efb3-4bac-931d-b53751136785

**Published:** 2013-08-06

**Authors:** Yixin Yang, Qianjin Liao, Fang Wei, Xiaoling Li, Wenling Zhang, Songqing Fan, Lei Shi, Xiayu Li, Zhaojian Gong, Jian Ma, Ming Zhou, Juanjuan Xiang, Shuping Peng, Bo Xiang, Hao Deng, Yunbo Yang, Yong Li, Wei Xiong, Zhaoyang Zeng, Guiyuan Li

The version of Figure 3 in the article is a duplicate of Figure 5.

The correct version of Figure 3 is available here: 

**Figure:**